# Effect of external use of Qingluo San on clinical efficacy in patients with acute gouty arthritis

**DOI:** 10.1186/s40001-022-00872-z

**Published:** 2022-11-11

**Authors:** Chun Shu, Fang Yang, Fubing Zhu, Dongping Hua

**Affiliations:** Department of Rheumatology, Tongling Hospital of Traditional Chinese Medicine, Anhui 244000 Tongling, China

**Keywords:** Gouty arthritis, Dampness–heat accumulation syndrome, Qingluo San

## Abstract

**Objective:**

The present study aimed to observe the clinical efficacy of the external use of Qingluo San combined with diclofenac sodium double-release enteric capsules in the treatment of acute gouty arthritis (dampness–heat accumulation syndrome).

**Methods:**

A total of 58 acute gouty arthritis patients were divided into two groups using the random number table method. Diclofenac sodium double-release enteric capsules were orally administered in the control group. Based on the treatment in the control group, the external use of Qingluo San was given in the treatment group, with 7-day course of treatment. The changes in visual analog scale (VAS) scores, the tenderness, swelling, and mobility function of the joint, and the traditional Chinese medicine (TCM) syndrome scores before and after the treatment, at day 0, 1, 3, 5 and 7, were observed in both groups, together with the comparison of laboratory indicators (erythrocyte sedimentation rate, ESR; C-reactive protein, CRP; uric acid, UA).

**Results:**

The total effective rate was 96.55% in the treatment group and 82.76% in the control group. After treatment, the VAS score, the tenderness, swelling and function scores of the joint, and the TCM syndrome scores decreased in both groups. The treatment group was superior in improving the VAS scores, the tenderness, swelling and mobility function of the joint, and TCM syndrome scores, when compared to the control group (*p* < *0.05*). The laboratory indicators, which included the ESR, CRP and UA, obviously decreased in both groups after treatment (*p* < *0.05*). The ESR significantly decreased in the treatment group, when compared to the control group (*p* < *0.05*).

**Conclusion:**

The combination of the external use of Qingluo San and oral administration of diclofenac sodium double-release enteric capsules can more rapidly relieve joint pain, and improve the clinical efficacy. This combination therapy also has certain advantages in relieving joint swelling and improving the mobility function of the joint. Hence, this is worthy of clinical promotion and application.

## Introduction

Acute gouty arthritis is an inflammatory reaction caused by the deposition of uric acid crystals around the joints due to a genetic or acquired decrease in uric acid excretion and/or impaired purine metabolism. The typical attacks usually start rapidly. These patients may often wake up in the middle of the night with foot pain that is cut or biting in nature, and present with redness, swelling, hotness and pain in the joint, and surrounding tissues. The site of the attack may involve multiple joints, especially the first metatarsophalangeal joint. With the improvement of the living standard and the change in diet structure, the incidence of gout in China has continuously increased annually, and tends to attack the younger population [1]. Therefore, it is especially important to find effective and safe treatment methods.

Qingluo Yin is composed of Sophora Flavescens Radix, Rhizoma Dioscoreae Tokoro, Green Wind Vine and Huangboon. It is the main prescription of Qingluo San, Qingluo Yin is aimed at clearing away heat and dampness, dredging collaterals and relieving arthralgia [2, 3]. Increasing of Fructus forsythiae, Rhubarb, Corydalis Rhizoma and Scorpion, they could add medicinal effect of the heat clearing and dehumidification, attack poison and disperse knot. These traditional Chinese medicines penetrate directly to the target joints through the skin, promoting the blood circulation in the affected area and effectively reduce the Erythrocyte sedimentation Rate (ESR) level, it improves the clinical symptoms of arthritis patients and the quality of life of patients.

The acute pain in gouty arthritis is often unbearable, and brings great physical and mental pain to the patient. Therefore, determining how to quickly and effectively relieve the pain and other symptoms has become a clinical focus. At present, the main means of Western medicine treatment are non-steroidal anti-inflammatory drugs (NSAIDs), colchicine, and glucocorticoids [4]. According to the theory of "transdermal" absorption, Chinese herbal medicine is externally applied to the skin, and absorbed into the body circulation through the stratum corneum by the capillaries, thereby exerting therapeutic effects.

In the present study, the investigators intended to treat patients with gouty arthritis using the comprehensive approach of Chinese and Western medicine by combining anti-inflammatory and analgesic treatment with the external use of traditional Chinese herbal medicine in the acute stage, and explore the therapeutic effect of the external use of Qingluo San combined with the oral administration of diclofenac sodium double-release enteric capsules, in the treatment of acute gouty arthritis.

## Clinical data and methods

### Study design

The present study was conducted in patients who were treated for acute gouty arthritis at Tongling Traditional Chinese Medicine Hospital from January 2018 to December 2019. It was an open-labeled, randomized and controlled clinical trial. All patients were divided into two groups using the random number table method: control group, received diclofenac sodium double-release enteric capsules for treatment; treatment group, received the external use of Qingluo San combined with diclofenac sodium double-release enteric capsules for treatment. The present study was approved by the Ethics Committee of Tongling Traditional Chinese Medicine Hospital. The enrolled patients provided a signed informed consent.

### Diagnostic criteria

The diagnosis of Western medicine for all the enrolled cases was referred to the diagnostic criteria for primary gouty arthritis in the 2016 China Gout Diagnosis and Treatment Guidelines [5]. Gout was classified according to 2015 ACR/EULAR gout classification standard. The diagnostic criteria for the dampness–heat syndrome in the Diagnostic Efficacy Criteria for Chinese Medical Evidence [6] and the Guidelines for Clinical Research on New Chinese Medicines (for Trial Implementation) [7] issued by the State Administration of Traditional Chinese Medicine in 2017 were mainly used in the diagnosis of the TCM syndrome. The manifestations for dampness–heat accumulation syndrome were, as follows: the small joints of the lower extremities were suddenly red, swollen and painful, refusing to be pressed with localized burning by touching and relief when cooled. The patient was accompanied by fever and thirst, restlessness and yellow urine. The tongue was red with a yellowish greasy coating. The pulse was fast and slippery.

### Inclusion criteria

Patients diagnosed according to “Diagnostic criteria” section: patients within 20–70 years old; patients with no treatment before admission, and were hospitalized within 72 h after disease onset; The patients and their families had informed consent, patients with high compliance, and provided a signed informed consent for the treatment.

### Exclusion criteria

Patients with severe liver and kidney diseases, cardiovascular and cerebrovascular diseases, respiratory diseases, and blood and endocrine diseases; patients with active gastrointestinal diseases; pregnant and lactating female patients; patients allergic to any of the drugs in the protocol.

### Therapeutic methods

The enrolled patients with gouty arthritis were divided into the treatment and control groups, according to a random number table, and both groups were given same basic non-pharmacological treatment, which mainly included the following: lifestyle modification, elevation of the affected limb, and low purine diet; prohibition of alcohol consumption (including beer, etc.); drinking > 2000 ml of water daily; avoiding strenuous exercise or exposure to cold, etc. In the control group, 75 mg of diclofenac sodium double-release enteric capsules were administered orally, twice daily (Tamler, Germany; import drug registration number: H20140548). In the treatment group, based on the treatment in the control group, Qingluo San was externally added to the target joints, and the drug comprised the following: Sophora Flavescens Radix (9 g), Huangboon (9 g), Green Wind Vine (9 g), Rhizoma Dioscoreae Tokoro (10 g), Fructus forsythiae (9 g), Rhubarb (9 g), Corydalis Rhizoma (9 g), and Scorpion (5 g); one dose daily. The external use of Chinese herbal medicine: the powdered Chinese herbal medicine was mixed with the adjuvants (70% black vinegar + 30% honey) to make the paste. Then, the paste was applied to the painful joints, with a thickness of approximately 4 mm (the scope of external application should exceed the scope of the lesion; if skin ulcer was present, the external application should be avoided). Gauze and external application of an elastic bandage was adopted for fixation. The external application was conducted for 6 h each time, once a day, for 7 days, as a course of treatment. 7 days was a course of treatment. At 0, 1, 3, 5 and 7 days before and after treatment, it was observed and compared between both groups changes of the visual analogue scale (VAS) scores, joint tenderness, joint swelling, joint activity function and TCM syndrome scores, uric acid (UA), C-reactive protein (CRP) and ESR. After the acute phase of gout, patients were started treatment with uric acid lowering in the study.

### Observation indicators

#### Primary observation indicators

The degree of pain of patients on treatment day 0, 1, 3, 5 and 7 was recorded using the VAS score (0–10 points): 0 point represented no pain and 10 points represented severe pain. The pain recording of the target joint was conducted by the patient, based on self-perception. The target joint was defined as the joint with the most severe pain, as reported by the patient at the time of enrollment.

#### Secondary observation indicators

The score for swelling and tenderness of the joint: on treatment day 0 and 7, the swelling and tenderness of the target joint in the patient were scored by the physician. The degree of joint swelling was scored using a 4-point scale: 0 = no swelling, 1 = palpable swelling, 2 = visible swelling, and 3 = swelling beyond the scope of the joint. The joint tenderness was scored using a 4-point scale: 0 = no tenderness, 1 = patient complained of tenderness, 2 = patient complained of tenderness and cowering, and 3 = patient complained of tenderness and avoidance.

The score for joint mobility function: no limitation to joint mobility = 0 point; limited mobility, but still able to perform normal activities = 1 point; significant limitation of mobility, and unable to perform general activities, but able to take care of themself = 2 points; felt pain during an activity, bedridden, and unable to take care of themself = 3 points.

The evaluation for total therapeutic efficacy, based on the criteria for determining the efficacy of Chinese medical syndrome in the Guidelines for Clinical Research on New Chinese Medicines: clinical cure: the disappearance or basic disappearance of TCM clinical symptoms and signs, and ≥ 95% reduction in the total score for the syndrome; significant effect: significant improvement in TCM clinical symptoms and signs after treatment, and ≥ 70% reduction in the score for the syndrome; effective: clinical symptoms and signs of TCM symptoms improved after treatment, with ≥ 30% reduction in the score for the syndrome; ineffective: no significant improvement in clinical symptoms and signs after treatment, or even presented with aggravation, with less than 30% reduction in the score for the syndrome. These effects were evaluated on the 7th day after treatment.

Observation of the efficacy of the TCM syndrome: the symptoms of joint swelling, joint pain, joint redness and heat, mobility function of the joint, fever, thirst and drinking, and yellow urine were scored before and after treatment, and the degrees were expressed according to the scoring method.

Laboratory examinations: the differences in the concentration of UA, CRP and ESR in the blood of patients in these two groups were evaluated before and after treatment.

#### Safety evaluation

The adverse reactions that occurred during the treatment, including gastrointestinal reactions, gastrointestinal hemorrhage, cardiovascular accidents, hemolysis, skin pruritus, redness and swelling, were recorded.

### Statistic analysis

The SPSS 22.0 software was adopted for the statistical analysis of the data. The measurement data were expressed as mean ± standard deviation. Paired *t*-test was used for the self-control comparison before and after treatment, and unpaired *t*-test was used for the comparison of two groups of data. If the data do not conform to the normal distribution, the nonparametric test is used. One-way ANOVA was used for multiple comparisons, and SNK *t*-test was used for multiple comparisons. *X*^2^-test was used for countable data, and rank-sum test was used for rank data. *p* < *0.05* was considered statistically significant.

## Results

### Comparison of the general data

From January 2018 to December 2019, a total of 58 patients, the observation and recording of symptoms and signs, testing of relevant laboratory indicators, evaluation of efficacy, and follow-up of the 58 patients were completed. There were 29 patients in the treatment group, in which 26 patients were male and three patients were female. The age of these patients ranged within 20–70 years old, with an average age of 54.66 ± 10.24 years old. There were 29 patients in the control group, in which 27 patients were male and two patients were female. The age of these patients ranged within 20–70 years, with an average age of 54.83 ± 11.79 years. There was no statistically significant difference between these two groups, in terms of the baseline characteristics of gender, age, pain score (VAS score), scores for joint swelling and joint tenderness, joint mobility function, and the TCM syndrome score (*p* > *0.05)*. The data were comparable (Table 1).

**Table 1:** Comparison of the general characteristics between the two groups

	Treatment group (*n* = 29)	Control group (*n* = 29)	*p*
Age (years, mean ± standard deviation)	54.66 ± 10.24	54.83 ± 11.79	0.953
Gender [n(%)]			
Male	26	27	1.000
Female	3	2	
History of gout (years, median [range])	3 (0–10)	3 (0–11)	0.856
BMI (kg/m^2^, mean ± standard deviation)	26.70 ± 4.07	26.20 ± 5.00	0.678
Uric acid (umol/L, before treatment)	506.28 ± 79.88	501.12 ± 95.49	0.824
VAS score (before treatment)	8.38 ± 0.97	8.34 ± 0.97	0.876
The score for joint swelling (before treatment)	5.93 ± 1.44	5.90 ± 1.63	0.941
The score for joint tenderness (before treatment)	7.03 ± 1.84	6.93 ± 1.81	0.836
The score for joint mobility function (before treatment)	2.45 ± 0.57	2.41 ± 0.50	0.777
The score for TCM syndrome (before treatment)	19.69 ± 4.18	19.55 ± 3.81	0.894

### The comparison of VAS scores before and after treatment between the two groups

The VAS pain scores gradually decreased in both groups after 1, 3, 5 and 7 days of treatment (Table 2). The decrease was significantly greater in the treatment group than in the control group (*p* < 0.05), and the VAS scores significantly improved after one day of treatment (*p* < 0.01).

**Table 2:** Comparison of VAS scores between the two groups before and after treatment

Group	Case	Before treatment	After treatment one day	After treatment three days	After treatment five days	After treatment seven days	Decrease range 7 days after treatment
Treatment group	29	8.38 ± 0.97	5.97 ± 1.18*^▽▽^	4.06 ± 1.41*^▽^	2.38 ± 1.78*^▽^	1.21 ± 2.06*^▽^	7.17^▽^
Control group	29	8.34 ± 0.97	6.93 ± 1.19	4.86 ± 1.46	3.55 ± 2.20	2.52 ± 2.71	5.82

Compared with before treatment *p < 0.05

Compared with the same period of treatment in the control group, ^▽^*p* < 0.05, ^▽▽^*p* < 0.01

### The comparison of efficacy of the TCM syndrome between the two groups

Compared with the total effective rate in the control group (82.76%), the total effective rate in the treatment group was 96.55%. The total effective rate in the treatment group was superior to that in the control group (Table 3).

**Table 3:** The comparison of therapeutic effects of the TCM syndrome before and after treatment between the two groups (%) 7 days after treatment

Group	Case	Clinical cure (%)	Significant effect (%)	Effective (%)	Ineffective (%)	Total effective rate (%)
Treatment group	29	8(27.59)	16(55.17)	5(17.24)	1(3.45)	28(96.55)
Control group	29	4(13.79)	8(27.59)	12(41.38)	5(17.24)	24(82.76)

Based on the rank-sum test: *Z* = − 2.579, *p* = 0.010 (*p* < 0.05)

### The comparison of the tenderness, swelling and mobility score of the joint, and TCM symptoms score between the two groups before and after treatment

Compared with the sores before the medication, the scores for joint tenderness, joint swelling, and joint mobility function were significantly lower after treatment, in both groups (*p* < 0.01), and the improvement was better in the treatment group, when compared to the control group (*p* < 0.05). This indicates that the local joint tenderness and swelling were significantly eliminated in the treatment group, and that the comprehensive TCM treatment can relieve local swelling and pain. Furthermore, there was a statistical difference in the improvement of the score for TCM syndrome in these two groups, between before and after treatment (*p* < 0.01). Moreover, the improvement in TCM syndrome score after medication was statistically significant in the treatment group, when compared to that in the control group (*p* < 0.01). This indicated that the improvement of the TCM syndrome was significantly superior in the treatment group, when compared to the control group (Table 4).

**Table 4:** The comparison of the scores for joint swelling and mobility function before and after treatment between the two groups ($\overline{X} \pm S$) 7 days after treatment

Items for evaluation	Treatment group (*n* = 29)		Control group (*n* = 29)	
	Before treatment	After treatment	Before treatment	After treatment
The score for joint tenderness	7.03 ± 1.84	1.96 ± 2.31*^▽^	6.93 ± 1.81	3.31 ± 3.04
The score for joint swelling	5.93 ± 1.44	1.52 ± 1.99*^▽^	5.90 ± 1.63	2.66 ± 2.27
The score for joint mobility function	2.45 ± 0.57	0.59 ± 0.78*^▽^	2.41 ± 0.50	1.03 ± 0.98
The score for the TCM syndrome	19.69 ± 4.18	4.93 ± 5.81*^▽▽^	19.55 ± 3.81	8.76 ± 6.99

Compared with before treatment by paired *t*-test within the group, ^*^p < 0.01; the difference between the two groups after treatment, by two independent samples *t*-test, ^▽^*p* < 0.05, ^▽▽^*p* < 0.01

### Comparison of the changes in laboratory indicators

ESR and CRP are commonly used to evaluate the activity of rheumatic diseases. Before treatment, there was no significant difference in serum levels of ESR, CRP and UA between the two groups (*p* > 0.05). After treatment, the serum ESR, CRP and UA decreased in both groups, and the difference was statistically significant (*p* < 0.01). ESR significantly decreased in the treatment group, and the difference was statistically significant, when compared to the control group (*p* < 0.05). However, there was no statistical significance in the difference in UA and CRP, between the treatment group and control group (*p* > 0.05). The results are presented in Table 5.

**Table 5:** Comparison of experimental indexes between the two groups before and after treatment ($\overline{X} \pm S$) 7 days after treatment

Item	Treatment group		Control group	
	Before treatment	After treatment	Before treatment	After treatment
ESR (mm/h)	45.41 ± 19.69	14.55 ± 8.79*^▽^	45.90 ± 25.16	24.72 ± 21.32*
CRP (mg/L)	31.51 ± 27.48	5.64 ± 6.02*	31.44 ± 31.61	9.56 ± 16.85*
UA (umol/L)	506.28 ± 79.88	438.98 ± 89.57*	501.12 ± 95.49	439.03 ± 64.49*

Compared with before treatment *p < 0.01; the difference between the two groups after treatment was statistically significant, two independent samples were tested by *t* test, ^▽^*p* < 0.05

### Comparison of the incidence of adverse events

During the treatment, three patients in the treatment group experienced stomachache or abdominal pain, two patients experienced itching and discomfort at the external application site, and one patient developed dizziness and headache. In the control group, four patients had stomachache or abdominal pain, and two patients had dizziness and headache. There was no statistical difference in the incidence of adverse events between the two groups (*p* > 0.05).

## Discussion

In this study, after treatment, the scores for joint tenderness, joint swelling, and joint mobility function were significantly lower, in both groups, and the improvement was better in the treatment group, when compared to the control group. The treatment group had a higher total effective rate and a more obvious improvement in TCM syndromes, the VAS score decreased more. The serum ESR, CRP and UA decreased in both groups. ESR decreased more significantly in treatment group.

Gout is a metabolic and inflammatory/immune disease, and the pathogenesis consists of two processes: uric acid in the form of urates, reaching levels sufficient to precipitate urates; the formation of crystals, and the inflammatory response to these formed crystals [8]. The most basic clinical pattern of gout is the acute onset of severe painful arthritis. This is characterized by redness, swelling and pain, with severe pain being the most initial symptom and the main factor for prompting the visit of patients [9].

In TCM, gouty arthritis belongs to the category of "paralysis-turbid and stagnant paralysis" and "chronic arthritis". The cause of the disease is mostly correlated with the consumption of fatty, sweet and thick food, alcohol abuse, external pathogen invasion, stagnation of the qi and blood, and blocking of the collateral by phlegm and blood stasis [10]. The external application of Chinese herbal medicine, which is topically applied to the affected area, has the advantages of being safe, simple and easily accepted by patients. Furthermore, this can avoid the possible hepatic "first-pass effect" and degradation of drugs in the gastrointestinal tract, which may occur with oral administration. Moreover, this eliminates the stimulating effect of drugs on the gastrointestinal tract, and the absorption of drugs is not affected by gastrointestinal factors. In addition, this is easy to use, and the drug administration can be interrupted anytime. The external application of Chinese herbal medicine for the treatment of gouty arthritis mainly works on the aspect of "clearing heat and dispelling dampness". Previous studies have shown that Chinese herbal medicine formulas, such as the external use of Sihuangshuimi San, can well-control the acute symptoms of gouty arthritis and reduce the chance of recurrence [11].

In the present study, the external application of Qingluo San combined with the oral administration of diclofenac sodium double-release enteric capsules was used for the treatment of acute gouty arthritis. The main ingredients of Qingluo San were, as follows: Sophora Flavescens Radix, Rhubarb, Corydalis Rhizoma, Fructus forsythiae, Huangboon, Green Wind Vine, Rhizoma Dioscoreae Tokoro, etc. Among these, the monarch herbal was Sophora Flavescens Radix. Sophora Flavescens Radix, which is bitter and cold in nature, has the function of clearing heat and detoxifying, dispelling wind and drying dampness, diuretic, etc. Previous studies have identified approximately 25 picrasidine. Among these, picrasidine and oxymatrine picrasidine have been shown to exert immunosuppressive and anti-inflammatory effects by inhibiting the proliferation of T cells [12, 13]. The minister herbals were Huangboon and Green Wind Vine. Huangboon, which has a cold nature and bitter taste, has the effect of clearing heat and drying dampness, removing fire and detoxifying swelling, and clearing deficiency heat. This has a unique curative effect in the clinical treatment of the dampness–heat syndrome. The Green Wind Vine is warm in nature and bitter in taste, which can dispel wind-dampness and relieve pain. The adjuvant Rhizoma Dioscoreae Tokoro is mild, bitter and sweet in nature, and is good for dispelling wind-dampness and paralysis. Pharmacological studies have shown that the active ingredients in the formula, such as Sophora Flavescens Radix, Rhizoma Dioscoreae Tokoro, Green Wind Vine and Huangboon, have anti-inflammatory and analgesic effects, and immunomodulatory effects, respectively [14, 15]. Rhubarb, which is bitter and cold in nature, has the effect of dipping heat and toxins, breaking down stagnation, and moving blood stasis. The effect is directly applied to the lesion, and the function of detoxifying and dispersing blood stasis, and relieving swelling and pain are more significant. Fructus forsythiae is bitter and slightly cold in nature, and can clear heat, detoxify, disperse nodules and reduce swelling. This has the name of “the Holy Medicine for those treating the Sore". After crushing the above drugs and applying these to the lesion, this can clear heat and remove paralysis. By directly penetrating the affected area through the skin, with the promotion of blood circulation and drug absorption in the affected area [16], the inflammatory swelling would be significantly reduced, the pain would be relieved, redness and swelling would subside, and the joint function would be rapidly restored. No allergic reactions, such as rash, were observed when used on a small scale, for symptoms in acute gout.

In the present study, there was no statistical significance in the difference in UA and CRP, between the treatment group and control group. This suggested that the transdermal treatment of herbal was better, when compared to the control group, in reducing the serum ESR. The combination of the external application of Qingluo San and oral administration of diclofenac sodium double-release enteric capsules more rapidly relieved the joint pain in the treatment group, and the decrease in VAS score in the treatment group was significantly greater than that in the control group. Meanwhile, the improvements observed for joint tenderness, joint swelling and joint mobility were superior in the treatment group, when compared to the control group, and the clinical efficacy was higher in the treatment group. There was no difference in the incidence of adverse events between these two groups. Therefore, the combination of the external use of Qingluo San and the oral administration of diclofenac sodium double-release enteric capsules can give full play to the characteristics of Chinese herbal medicine, which is "simple, convenient and low-priced", and has the advantages of safety, simplicity and visibility. This could achieve significant clinical results in the short term. The present therapy is a special treatment for the treatment of turbid paralysis in Chinese herbal medicine, which is not only effective, but also safe and easily accepted by patients [17].

There are some limitations in this study. First, the study has fewer participants and is a single center study, which may cause bias in the results. Secondly, this study did not carry out long-term follow-up and did not observe long-term efficacy.

## Concluison

In summary, the combination of the external use of Qingluo San and the oral administration of diclofenac sodium double-release enteric capsules can give full play to the characteristic advantages of Chinese herbal medicine. This can meet clinical treatment needs, and is worthy of further clinical promotion and application.

## Data Availability

The datasets used and analyzed during the current study are available from the corresponding author on reasonable request.
